# Dihydrotanshinone I Attenuates Atherosclerosis in ApoE-Deficient Mice: Role of NOX4/NF-κB Mediated Lectin-Like Oxidized LDL Receptor-1 (LOX-1) of the Endothelium

**DOI:** 10.3389/fphar.2016.00418

**Published:** 2016-11-08

**Authors:** Wenwen Zhao, Chunxia Li, Hongwei Gao, Qin Wu, Jingshan Shi, Xiuping Chen

**Affiliations:** ^1^State Key Laboratory of Quality Research in Chinese Medicine, Institute of Chinese Medical Sciences, University of MacauMacao, China; ^2^Key Lab for Pharmacology of Ministry of Education, Department of Pharmacology, Zunyi Medical CollegeZunyi, China

**Keywords:** dihydrotanshinone I, lipopolysaccharide, LOX-1, ROS, atherosclerosis

## Abstract

Dihydrotanshinone I (DHT) is a natural compound extracted from *Salvia miltiorrhiza* Bunge which has been widely used for treating cardiovascular diseases. However, its role in atherosclerosis remains unclear. In this study, the effect of DHT on atherosclerosis were investigated using apolipoprotein E-deficient (ApoE^-/-^) mice and endothelial cells. In lipopolysaccharide (LPS)-stimulated human umbilical vein endothelial cells (HUVECs), DHT (10 nM) decreased lectin-like ox-LDL receptor-1 (LOX-1) and NADPH oxidase 4 (NOX4) expression, reactive oxygen species (ROS) production, NF-κB nuclear translocation, ox-LDL endocytosis and monocytes adhesion. Silence NOX4 inhibited LPS-induced LOX-1 expression, NF-κB nuclear translocation, ox-LDL endocytosis and monocytes adhesion. In ApoE^-/-^ mice fed with an atherogenic diet, DHT (10 and 25 mg kg^-1^) significantly attenuated atherosclerotic plaque formation, altered serum lipid profile, decreased oxidative stress and shrunk necrotic core areas. The enhanced expression of LOX-1, NOX4, and NF-κB in aorta was also dramatically inhibited by DHT. In conclusion, these results suggested that DHT showed anti-atherosclerotic activity through inhibition of LOX-1 mediated by NOX4/NF-κB signaling pathways both *in vitro* and *in vivo*. This finding suggested that DHT might be used as a potential vascular protective candidate for the treatment of atherosclerosis.

## Introduction

Atherosclerosis is considered to be a chronic inflammatory disease and plays a key role in heart attacks, strokes, and peripheral vascular diseases (Libby, 2002; Frostegard, 2013). Over the last decades, atherosclerotic cardiovascular diseases remain the leading cause of mortality worldwide (Gaziano et al., 2010). One critical event in the initiation of atherosclerosis is the ox-LDL uptake, which contributes to the atherosclerotic foam cell and plaque formation (Steinberg, 1997; Kita et al., 2001). Ox-LDL uptake is principally mediated by a variety of specific receptors including SR-A I/II, CD36, SR-BI, etc. (Li and Glass, 2002; Hu et al., 2010). Recently, LOX-1 has been identified as the main endothelial receptor for ox-LDL (Sawamura et al., 1997; Chen et al., 2002). LOX-1 is a 50-kD transmembrane protein highly expressed on macrophages, vascular smooth muscle cells, and especially endothelial cells, which plays important roles in the pathogenesis of diabetic cardiovascular complications, myocardial ischemia, and atherosclerosis (Lu and Mehta, 2011; Yan et al., 2011; Xu et al., 2013b). Disturbance of LOX-1 mediated signaling pathways has been proposed as a potential strategy for the anti-atherosclerotic drug discovery (Sawamura et al., 1997; Chen et al., 2002, 2007). Although a variety of cells, including monocytes, dendritic cells, lymphocytes, eosinophils, mast cells and smooth muscle cells, contribute to atherogenesis (Moore et al., 2013), endothelial dysfunction is considered to be an early marker and played important roles in the pathophysiology of atherosclerosis (Davignon and Ganz, 2004). ROS, an important second messenger, is linked to many vascular diseases including atherosclerosis (Vara and Pula, 2014). NOX4 is an important source of ROS in endothelial cells (Ray and Shah, 2005). However, the role of NOX4 in atherosclerosis is controversial (Hu et al., 2008; Zhao et al., 2014). Furthermore, the TLRs-NF-κB pathway has been reported to participate in the anti-atherosclerotic effect of several natural products such as quercetin, pycnogenol, and procyanidins (Martinez-Micaelo et al., 2012; Luo et al., 2015; Bhaskar et al., 2016).

*Salvia miltiorrhiza* Bunge (Danshen) is a famous medical herb with a long history of clinical application for treating cardiovascular diseases, cancer, and osteoporosis (Cao et al., 2012; Chen et al., 2014; Guo et al., 2014). Its ingredients have been widely explored since last century. Two groups of ingredients, the hydrophilic phenolic acids and the lipophilic tanshinones, have been identified as the main bioactive components. Tanshinones are a group of abietane diterpenes composed of four rings. Totally, more than 50 tanshinones have been isolated and identified since 1934 (Fan et al., 2012). However, only a few of them, such as tanshinone IIA (Xu and Liu, 2013a) and cryptotanshinone (Chen et al., 2013), have been widely investigated. DHT (Supplementary Figure S2), a tanshinone isolated from Danshen, is structurally similar to that of tanshinone IIA and cryptotanshinone. For a long time, investigation to its bioactivities was mainly focused on its cytotoxicity to a variety of tumor cells (Liu et al., 2015; Wang et al., 2015). Although increasing evidence suggested that DHT has beneficial potentials for the treatment of cardiovascular diseases (Zhang et al., 2009; Liu et al., 2010), its anti-atherosclerotic effect, either *in vivo* or *in vitro*, has not been fully established. Here, this effect was evaluated with experimental atherosclerosis model in ApoE^-/-^ mice and HUVECs. Furthermore, the underlying mechanisms were explored as well.

## Materials and Methods

### Materials

DHT (purity ≥ 98% by HPLC analysis, a lipophilic diterpenes) was purchased from Chengdu Herbpurify Co. Ltd. (Chendu, China). Hoechst 33342, *N*-acetyl cysteine (NAC), DPI, Rot, TTFA, AA, All, NDGA, LPS (*Escherichia coli* serotype 055:B5, LPS), 5-(6)-carboxy-2′,7′-dichlorodihydrofluorescein diacetate (DCFH_2_-DA), PDTC, 2,2-Diphenyl-1-picrylhydrazyl (DPPH) and lucigenin were purchased from Sigma–Aldrich (St. Louis, MO, USA). Lipofectamine TM2000, DHE and Amplex Red were purchased from Life Technology (Grand Island, NY, USA). Antibodies for NOX4, p22-phox, TLR4, MyD88 were purchased from Santa Cruz Biotechnology (Santa Cruz, CA, USA). Antibodies for NF-κB p65, GAPDH, and Histone H3 were purchased from Cell Signaling Technology (Beverly, MA, USA). Anti-LOX-1 antibody was obtained from R&D (Minneapolis, MN, USA). The BCA protein kits were purchased from Thermo Fisher (Suwanee, GA, USA). Fraction-PREP Cell Fractionation Kit was purchased from BioVision (Milpitas, CA, USA). SiRNA for TLR4, MyD88, and NOX4 were purchased from Gene Pharma Company (Shanghai, China). Primers and other materials for real-time PCR were purchased from Sangon Biotech (Haimen, China) and TaKaRa Bio Group (Shiga, Japan). DiI-ox-LDL was purchased from Yiyuan Biotechnology (Guangzhou, China).

Kits for TC, LDL-C, HDL-C, TG, MDA, GSH, and SOD were obtained from Nanjing Jiancheng Bioengineering Research Institute (Nanjing, China).

### Cell Culture

Human umbilical vein endothelial cells were cultured in Vascular Cell Basal Medium with Endothelial Cell Growth Kit-BBE at 37°C in a humidified atmosphere of 5% CO_2_. Before passaging cells, issue culture flasks, 96-well plates and 6-well plates were pre-coated with 0.1% gelatin. All assays were conducted using low cell passage cells (2–5 passages).

Human monocyte cell line (THP-1) obtained from ATCC (no: TIB-202, Rockville, MA, USA) was cultured in RPMI 1640 medium containing 10% fetal calf serum, 2 mM glutamine, 100 U/mL penicillin, and 100 μg/mL streptomycin. Cells were seeded at 37°C in a humidified atmosphere of 5% CO_2_ and 95% air.

### Animal Experiment

Male ApoE^-/-^ mice (6–8 weeks old) on C57BL/6J background and age-matched wild-type C57BL/6J controls were purchased from Beijing HFK Bioscience Co., Ltd. (Beijing, China). Mice were housed in SPF-grade animal facilities (Certificate no. SCXK 2014-0004) at Zunyi Medical College, with a 12 h light/dark cycle, at 23°C (±2°C). All animal procedures follow the NIH guide for the Care and Use of Laboratory Animals (NIH Publications No. 80-23, revised 1978), and approved by the Institutional Animal Use and Care Committee of Zunyi Medical College. Starting from 6 weeks, the mice were fed with a HCD (54.35% raw grain, 20% lard, 0.15% cholesterol, 15% sucrose, 0.5% Sodium Cholate, 10% yolk powder) for 12 weeks. All ApoE^-/-^ mice were dosed daily *via* intragastric gavage with 10 and 25 mg kg^-1^ DHT dissolved in 0.5% CMC-Na or administered 0.5% CMC-Na alone (vehicle control) (*n* = 8 per group).

### Immunofluorescence Assay

Cells (1 × 10^4^ cells/well) were seeded on glass slides in 96-well plates. After LPS treatment (Sigma–Aldrich, St. Louis, MO, USA) (with or without DHT pretreatment), the slides were fixed with 4% PFA for 30 min. Then the slides were permeabilized with PBS-T (containing 0.1% Triton x-100 in PBS solution) and blocked with PBS-B (containing 4% BSA in PBS solution). After incubated with primary antibody (1:1000) and secondary antibody (1:2000), cells were stained with Hoechst 33342 in dark for 5 min. The protein location and expression were observed with IN Cell Analyzer 2000 (GE Healthcare).

### Detection of Reactive Oxygen Species, Superoxide Anion ($O_{2}^{•-}$), and Hydrogen Peroxide (H_2_O_2_)

The effect of DHT on LPS-induced $O_{2}^{•-}$, H_2_O_2_, and ROS production were measured by DHE, Amplex Red, and DCFH_2_-DA, respectively. Briefly, after LPS treatment for 30 min, cells were washed with PBS and incubated with DHE (10 μM), Amplex Red (50 μM), or DCFH_2_-DA (10 μM) for 30 min in the dark at 37°C. Then, cells were washed with PBS for three times and fluorescence density was measured by Spectra Max M5 Microplate Reader (Molecular Devices, Sunnyvale, CA, USA) or by a flow cytometry using a FACSCantoTM system (BD Biosciences).

### DPPH Assay

To evaluate the direct scavenging effect of DHT on free radicals in cell-free system, the DPPH system was adapted. DHT (0.01–1 μM, final concentration) was incubated with ethanol solution of DPPH (5.0 × 10^-4^ M). After thoroughly mixing, the solutions were kept in the dark for 20 min and the absorbency was measured at 517 nm.

### Western Blotting

Treated HUVECs were washed twice with ice-cold PBS and lysed with RIPA buffer supplemented with a protease cocktail and phosphatase inhibitors. The cell lysates were separated using 8–12% SDS-PAGE and transferred onto PVDF membranes. After blocked with 5% non-fat milk in TBST (20 mM Tris-HCl, 500 mM NaCl, and 0.1% Tween 20) at room temperature for 1 h, membranes were incubated with specific primary antibodies and secondary antibodies. The protein-antibody complexes were detected by ECL Advanced Western Blot detection Kit. The intensity of the band was quantitated with Quantity One software (Bio-Rad).

### Preparation of Membrane, Cytoplasmic and Nuclear Fractions

The cytoplasmic and membrane proteins were isolated using Fraction-PREP cell fractionation kit (Biovision Inc., Milpitas, CA, USA) according to the manufacturer’s instructions^1^.

### SiRNA Transfection

Gene silencing experiment of TLR4, MyD88, and NOX4 with siRNA was performed according to our previous report (Zhao et al., 2014).

### Real-Time RT-PCR

The mRNA expression of LOX-1 was determined with real-time PCR as our previous report (Zhao et al., 2014).

### DiI-ox-LDL Uptake

The DiI-ox-LDL uptake assay was performed according to our previous study with minor revisions (Zhang et al., 2006). Cells (1.0 × 10^4^/well) in 96-well plate cultured for overnight were pretreated with DHT, inhibitors, or anti-LOX-1 antibody for 1 h and then co-treated with LPS for another 24 h. After incubated with DiI-ox-LDL (1 μg/mL) for 1 h at 37°C in the dark, the cells were gently washed with PBS for three times to remove the free DiI-ox-LDL and the ox-LDL uptake was measured with IN Cell Analyzer 2000.

### Monocyte-endothelial Cell Adhesion Assay

After various treatments, HUVECs were co-incubated with THP-1 cells labeled with Hoechst 33342 for 3 h in the dark at 37°C. The non-adherent THP-1 cells were washed gently with PBS. Images were obtained under random fields of each well using IN Cell Analyzer 2000.

### Immunohistochemistry

Immunohistochemical analysis on aortic sinus was performed as described previously in detail (Xu et al., 2011). Sections, 8 μm thick, were used for immunohistochemical staining with LOX-1, NOX4 and phosphorylated NF-κB p65 antibodies. Color reaction was developed with diaminobenzidine.

### Aorta Collection and Lesion Size Evaluation

To evaluate plaque extension, frozen sections of the aortic sinus (8 μm) were stained using Oil Red O, hematoxylin and eosin (H&E), respectively. Related experiments were performed following the method of Paigen et al. (1987).

### Blood Parameters

Blood samples were collected from the orbit before the mice were killed. Serum TC, LDL-C, HDL-C, and TG were measured using standard enzymatic colorimetric assays. MDA, GSH, and SOD were detected by commercial kits.

### Statistical Analysis

Twenty four ApoE^-/-^ mice were randomly allocated to groups and equal group sizes were obtained (*n* = 8 per group). Data were expressed as the means ± SD. The differences between groups were analyzed using Prism 5.0 (Graph Pad Software Inc, San Diego, CA, USA) and the statistical analysis was performed by analysis of variance (one-way ANOVA) followed by Student Newman–Keuls test. *p* < 0.05 is considered statistically significant.

## Results

### DHT Inhibited LPS-Induced LOX-1 Expression

**FIGURE 1:**
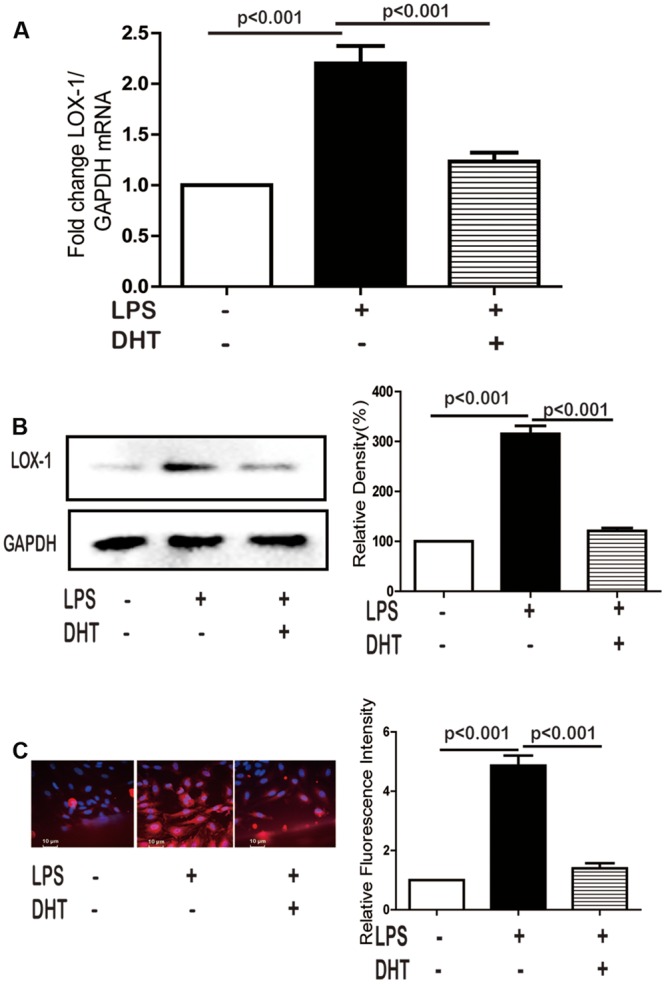
**DHT inhibited LPS-induced LOX-1 expression.** Cells were pretreated with DHT (10 nM) for 1 h and then stimulated with LPS (5 μg/mL) for 24 h, the LOX-1 mRNA **(A)**, protein **(B)**, and localization **(C)** were detected by real-time PCR, western blotting and immunofluorescence (60×), respectively. DHT, dihydrotanshinone I.

Firstly, the cytotoxic effect of DHT on HUVECs was determined by MTT assay. Results showed that DHT was cytotoxic to HUVECs at 1 μM (Supplementary Figure S1). To minimize the cytotoxic effect of DHT on cell viability, 10 nM DHT was chosen for further study. Compared with untreated HUVECs, 24 h stimulation with LPS induced significant LOX-1 expression at both mRNA (**Figure 1A**) and protein (**Figure 1B**) levels, which were dramatically inhibited by DHT pretreatment. Furthermore, immunofluorescence results showed that LPS-induced LOX-1 expression was mainly localized on the membrane, which was significantly suppressed by DHT as well (**Figure 1C**).

### DHT Inhibited LPS-Induced LOX-1 Expression via TLR4/Myd88

**FIGURE 2:**
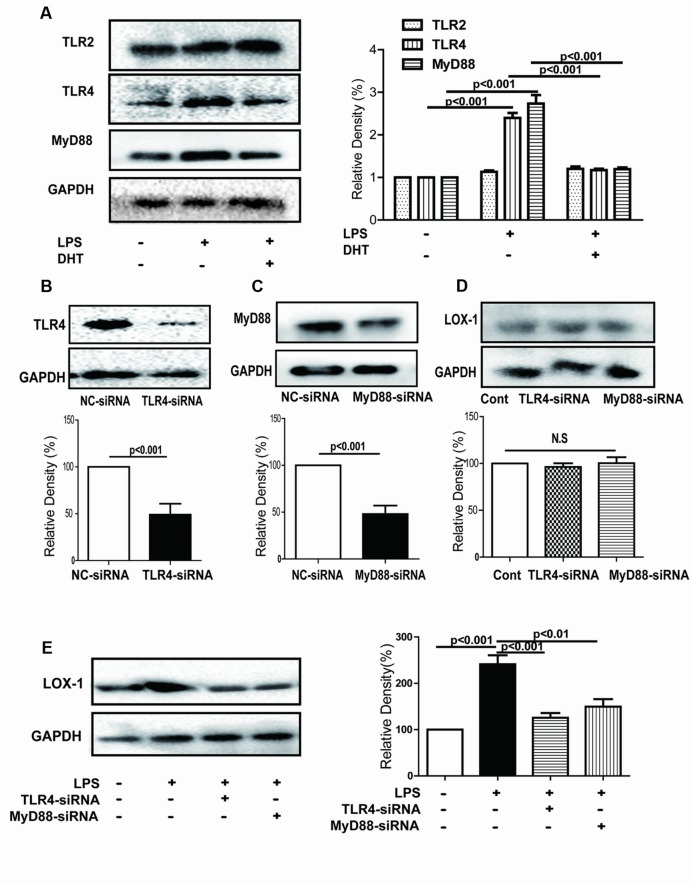
**DHT inhibited LPS-induced LOX-1 expression *via* TLR4/MyD88.** Cells were pretreated with DHT (10 nM) for 1 h and then stimulated by LPS (5 μg/mL) for 24 h, expressions of TLR2, TLR4 and MyD88 were determined by western blotting **(A)**. Cells were transfected with siRNAs for TLR4 **(B)** and MyD88 **(C)** and then stimulated with LPS (5 μg/mL) for 24 h and the LOX-1 expression were determined by western blotting **(D,E)**. Cont, control; NC-siRNA, negative control siRNA. DHT, dihydrotanshinone I; N.S, no significant differences.

Our previous study demonstrated that TLR4/MyD88 signal pathway was involved in LPS-induced LOX-1 expression in endothelial cells (Zhao et al., 2014). Here, LPS-induced expression of TLR4 and MyD88 was obviously inhibited by DHT (**Figure 2A**). Furthermore, silence of either TLR4 or MyD88 did not affect the expression of LOX-1 in the absence of LPS (**Figures 2B–D**). In addition, the LPS-induced expression of LOX-1 was significantly decreased in both TLR4 and MyD88 silenced cells (**Figure 2E**).

### DHT Inhibited LPS-Induced ROS Generation

**FIGURE 3:**
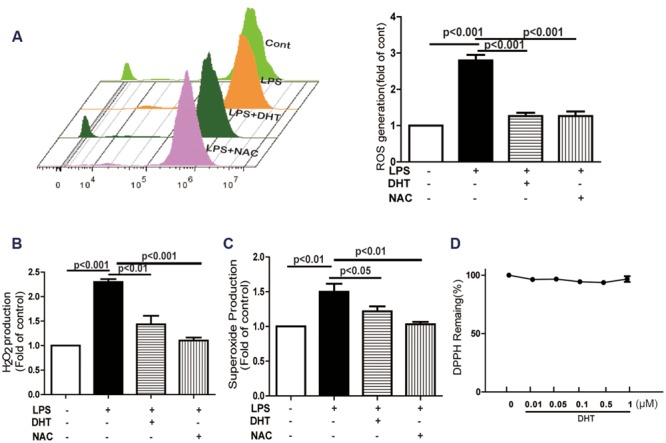
**DHT inhibited LPS-induced ROS, H_2_O_2_, and $O_{2}^{•-}$ production.** ROS **(A)**, H_2_O_2_ **(B)**, and **$O_{2}^{•-}$ (C)** production were determined by DCFH_2_-DA, Amplex Red and DHE respectively, after LPS (5 μg/mL) treatment for 1 h with or without DHT (10 nM) or NAC (5 mM) pretreatment for 1 h. Effect of DHT on DPPH radical was determined in a cell-free system **(D)**. DHT, dihydrotanshinone I; ROS, reactive oxygen species; NAC, *N*-acetyl cysteine.

LOX-1 was reported to be upregulated by oxidative stress in vascular smooth muscle cells (Sun and Chen, 2011) and endothelial cells (Zhao et al., 2014). LPS treatment for 4 h induced endothelial ROS generation as detected by DCFH_2_-DA (**Figure 3A**). Amplex Red and DHE assay showed that the generation of both H_2_O_2_ and $O_{2}^{•-}$ was also significantly increased after LPS stimulation. DHT and NAC pretreatment dramatically inhibited these increases (**Figures 3B,C**). However, no effect of DHT on DPPH was observed (**Figure 3D**).

### DHT Inhibited LPS-Induced LOX-1 Expression Through NOX4

**FIGURE 4:**
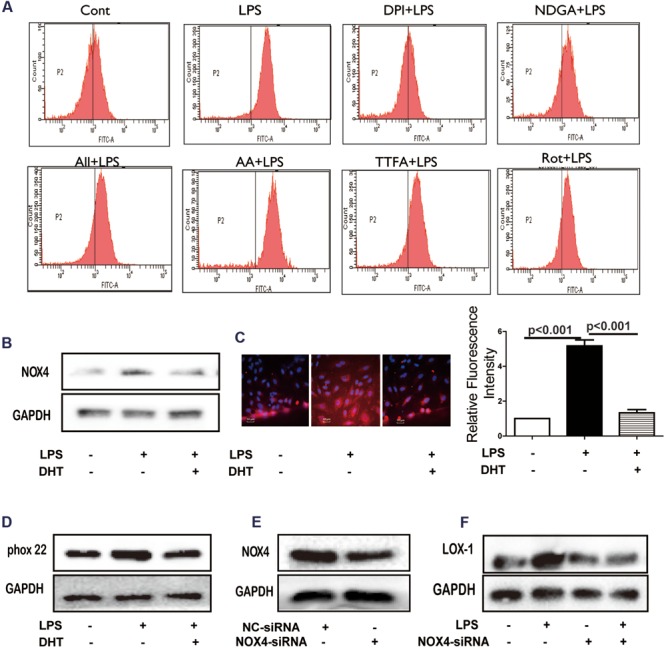
**DHT inhibited LPS-induced NOX4 dependent ROS generation.** Cells were treated with LPS (5 μg/mL) with or without 1 h pretreatment with DPI (1 μM), NDGA (10 μM), All (10 μM), Rot (20 μM), TTFA (10 μM) or AA (5 μM) and the ROS was determined **(A)**. NOX4 expression and localization were determined by western blotting **(B)** and immunofluorescence **(C)** (60×). P22phox expression was determined by western blotting **(D)**. Cells were transfected with NOX4 siRNA, NOX4 **(E)** and LOX-1 expression **(F)** were determined by western blotting. Cont, control group; DHT, dihydrotanshinone I; NC-siRNA, negative control siRNA; DPI, diphenyleneiodonium chloride; NDGA, nordihydroguaiaretic acid; All, allopurinol; AA, antimycin A; Rot, rotenone; TTFA, 2-thenoyltrifluoroacetone.

LPS-induced ROS generation was significantly inhibited by DPI while NDGA, allopurinol (All), Rot, TTFA, and AA showed no obvious effect (**Figure 4A**). Furthermore, LPS increased NOX4 protein expression (**Figure 4B**) and membrane aggregation (**Figure 4C**) which was also inhibited by DHT. LPS-induced p22phox expression was suppressed by DHT (**Figure 4D**). In addition, silence NOX4 (**Figure 4E**) dramatically inhibited LPS-induced LOX-1 expression (**Figure 4F**) (Supplementary Figure S3).

### DHT Inhibited LPS-Induced LOX-1 Expression by Suppressing NF-κB Activation

**FIGURE 5:**
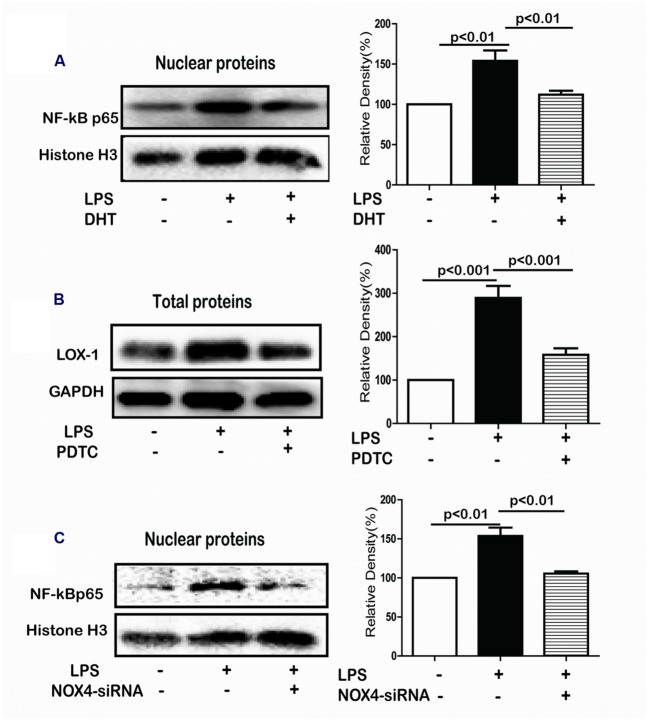
**DHT inhibited LPS-induced LOX-1 expression *via* NF-κB.** Cells were treated with LPS (5 μg/mL) for 24 h with or without pretreatment with DHT, PDTC, or NOX4 siRNA and the expression of p65 **(A)**, LOX-1 **(B)**, and p65 **(C)** was detected by western blotting. DHT, dihydrotanshinone I; PDTC, pyrrolidine dithiocarbamic acid.

NF-κB pathway plays an essential role in LPS-induced LOX-1 expression in HUVECs (Zhao et al., 2014). The NF-κB p65 in the nuclear in response to LPS was significantly increased which was remarkably suppressed by DHT treatment (**Figure 5A**). Furthermore, LPS-induced LOX-1 expression was inhibited by PDTC, a NF-κB inhibitor (**Figure 5B**). In addition, LPS-induced p65 nuclear translocation was significantly suppressed by NOX4 siRNA (**Figure 5C**).

### DHT Inhibited LPS-Induced Ox-LDL Uptake Mediated by LOX-1

**FIGURE 6:**
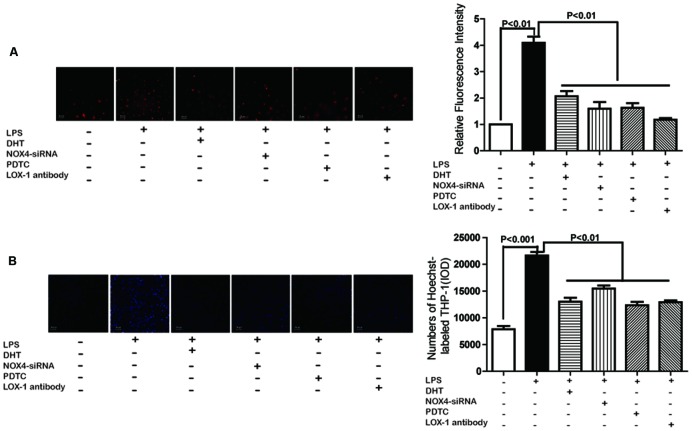
**DHT inhibited LPS-induced ox-LDL uptake and monocyte adhesion mediated by LOX-1.** Cells were pretreated with DHT (10 nM), siNOX4, PDTC (10 μM), or anti-LOX-1 Ab (10 μg/mL) for 1 h before LPS treatment for 24 h. Then cells were incubated with DiI-ox-LDL (5 μg/mL) and the ox-LDL uptake was measured with an inverted fluorescent microscopy (20×) **(A)**. Cells received same treatment and were co-incubated with Hoechst 33342 labeled THP-1 cells. The attached THP-1 cells were visualized by an inverted fluorescent microscopy (20×) **(B)**. DHT, dihydrotanshinone I; NC-siRNA, negative control siRNA; PDTC, pyrrolidine dithiocarbamic acid.

In the DiI-ox-LDL uptake assay, compared with untreated cells, LPS stimulation resulted in increased intracellular red fluorescence in stimulated endothelial cells suggesting the increase of DiI-ox-LDL internalization (**Figure 6A**). This increase was significantly reversed by pretreatment with DHT, NOX4 siRNA, PDTC, and anti-LOX-1 antibody.

### DHT Inhibited LPS-Induced THP-1 Adhesion Mediated by LOX-1

We previously reported the potential role of LOX-1 in LPS-mediated monocyte-endothelial cells adhesion (Zhao et al., 2014). In present research, LPS treatment for 24 h significantly increased THP-1 cell adhesion to endothelial cells, which was also dramatically inhibited by DHT, NOX4 siRNA, PDTC, and anti-LOX-1 antibody (**Figure 6B**).

### DHT Ameliorated the Serum Lipid Profile in ApoE^-/-^ Mice

**FIGURE 7:**
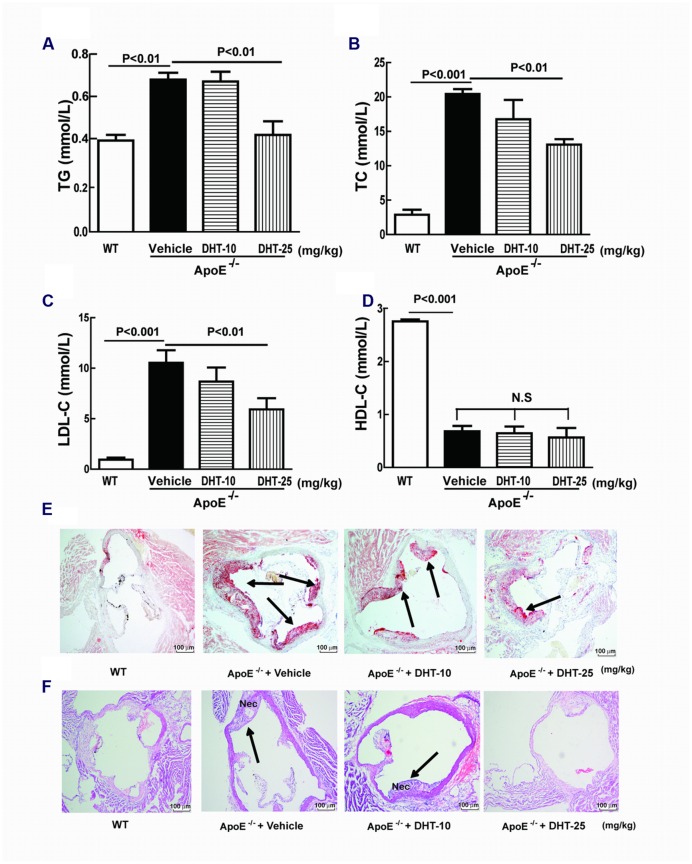
**DHT ameliorated serum lipid profile and reduced atherosclerosis in ApoE^-/-^ mice.** The serum TG, TC, LDL-C, and HDL-C were measured by colorimetric assays **(A–D)**. Aortic sinus sections were stained with Oil Red O and H&E to detect plaque sizes **(E)** (40×) and necrotic core areas **(F)** respectively (40×). DHT, dihydrotanshinone I; TC, total cholesterol; TG, triglycerides; LDL-C, LDL cholesterol; HDL-C, HDL- cholesterol; Nec, necrotic core area; WT, wild type; ApoE^-/-^, apolipoprotein E-deficient; N.S, no significant differences.

As shown in **Figures 7A–C**, compared with the WT mice, high-fat diet induced dramatically increase in TG, TC, LDL-C, as well as HDL-C in ApoE^-/-^ mice. Low dosage of DHT mildly decreased the lipid levels of TC and LDL-C but showed no effect on TG while at high dosage DHT dramatically inhibited TG, TC, and LDL-C. Both dosages of DHT showed no effect on HDL-C (**Figure 7D**).

### DHT Reduced Atherosclerotic Plaque Development in ApoE^-/-^ Mice

Histopathological studies had showed that an atherosclerotic plaques (Oil red O-stained red area) was formed and obvious necrotic core areas (H&E-stained area) were observed in ApoE^-/-^ mice. DHT administration significantly reduced plaque sizes as well as shrank necrotic core areas. High dosage showed more potent inhibitory effect (**Figures 7E,F**).

### DHT Reduced Oxidative Stress in ApoE^-/-^ Mice

**FIGURE 8:**
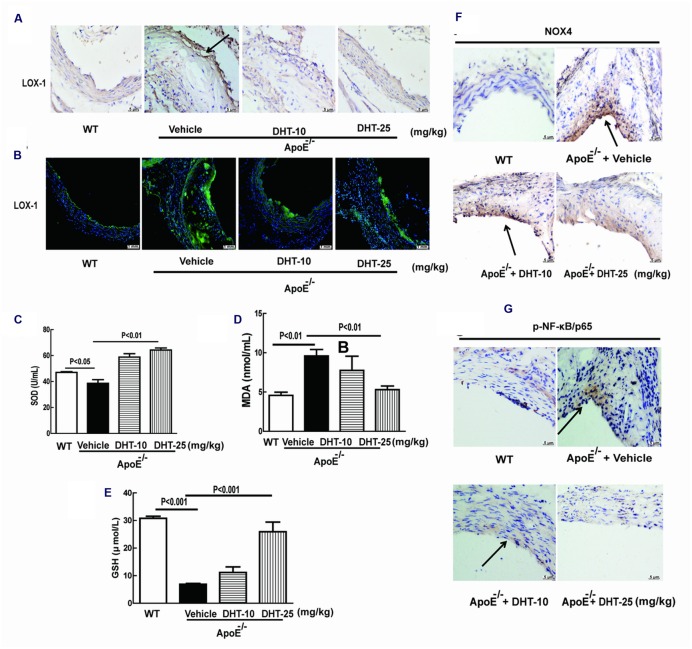
**DHT reduced oxidative stress, inhibited LOX-1, NF-κB, and LOX-1 expression in ApoE^-/-^ mice.** Expression of LOX-1 **(A)**, NOX4 **(F)**, and NF-κB p65 **(G)** in the aorta was detected by immunohistochemistry. The MDA, GSH and SOD levels were detected by kits **(C–E)**. LOX-1 expression was detected by immunofluorescence **(B)**. DHT, dihydrotanshinone I; MDA, malondialdehyde; GSH, glutathione; SOD, superoxide dismutase; WT, wild type; ApoE^-/-^, apolipoprotein E-deficient.

As shown in **Figures 8C–E**, there was a slight decrease of SOD while nearly fivefolds decrease of GSH and twofolds increase of MDA in ApoE^-/-^ mice compared to WT mice. The decreased SOD and GSH and the increased MDA levels in the serum were significantly reversed by DHT administration.

### DHT Inhibited NOX4, NF-κB and LOX-1 Expression in ApoE^-/-^ Mice

Immunohistochemistry showed that LOX-1 was expressed at low level in aorta of WT mice but significantly increased in ApoE^-/-^ mice, which was dramatically decreased in DHT treated mice (**Figure 8A**). LOX-1 was mainly expressed on the endothelial cells, which was further confirmed by immunofluorescence (**Figure 8B**). Furthermore, immunohistochemistry analysis of aortic sinus showed that the expression of NOX4 and phosphorylated NF-κB p65 were remarkably increased in ApoE^-/-^ mice, which were significantly inhibited by DHT as well (**Figures 8F,G**).

## Discussion

Herb extracts and single compounds from *Salvia miltiorrhiza*, *Curcuma longa*, *Rheum undulatum*, and *Panax notoginseng*, have been reported to regulate multiple targets that involved in the initiation and progress of atherosclerosis (Quiles et al., 2002; Fan et al., 2012; Zeng et al., 2012). Here, we showed that DHT isolated from *Salvia miltiorrhiza* Bunge significantly attenuated atherosclerosis through regulating LOX-1 expression both *in vivo* and *in vitro*. The main findings of this study are: (1). DHT inhibited LPS-induced LOX-1 expression through TLR4/NOX4/NF-κB pathway in endothelial cells. (2). DHT benefited atherosclerosis by inhibiting LOX-1-mediated ox-LDL endocytosis and monocyte-endothelial cells adhesion in endothelial cells. (3). DHT attenuated atherosclerosis in ApoE^-/-^ mice through regulating LOX-1 expression.

Our previous study demonstrated that LPS induced LOX-1 expression in a TLR4/MyD88-dependent manner (Zhao et al., 2014). Here, we found that LPS-induced expression of TLR4 and MyD88 was dramatically inhibited by DHT. However, DHT showed no effect on TLR2 expression. Furthermore, silencing of TLR4 and MyD88 significantly reversed LPS-induced LOX-1 expression. Thus, DHT might inhibit LPS-induced LOX-1 expression in a TLR4/MyD88 manner.

TLR4/MyD88 functions as the upstream of ROS (Zhang et al., 2009). Cryptotanshinone, an analog of DHT, has been reported to inhibit ox-LDL- or LPS- induced endothelial ROS formation (Chen et al., 2008; Zhao et al., 2014). In present study, DHT significantly inhibited LPS-induced ROS production in endothelial cells. Further investigation showed that DHT inhibited both LPS-induced $O_{2}^{•-}$ and H_2_O_2_ production but has no effect on DPPH free radical. Thus suggested that DHT may not be a ROS scavenger. Since LPS-induced ROS production was potentially inhibited by NDAPH oxidase inhibitor DPI but not by LOX inhibitor NDGA, XO inhibitor ALL, mitochondria respiratory electron-transport chain inhibitors AA, TIFA and Rot, it suggested that NADPH oxidase was the main source of ROS in response to LPS. Among the 7 NADPH oxidase isoforms NOX4 was identified as the main contributor for LPS-induced ROS in HUVECs (Mehta et al., 2006; Park et al., 2006; Zhao et al., 2014). In our study, DHT suppressed LPS-induced NOX4 expression and membrane translocation. Previous studies showed that activated TLR4/MyD88 upregulated NOX4 expression in LPS-activated cells and lead to atherosclerosis (Patel et al., 2006; Petry et al., 2006). Consistent with these findings, here we found that silence TLR4 decreased NOX4 expression in response to LPS in endothelial cells (data not shown). Furthermore, DHT inhibited LPS induced p22phox, an important NOX4 adaptor for NOX4 activation (Geiszt and Leto, 2004), expression. In addition, silence NOX4 inhibited LPS-induced LOX-1 expression. Collectively, these results suggested that DHT inhibited LPS-induced LOX-1 expression mediated by NOX4-dependent ROS.

The transcription factor NF-κB plays an essential role in regulating of various inflammatory cytokines secretion mediated by the intracellular ROS levels (Tak and Firestein, 2001). Increased nuclear translocation of NF-κB p65 by LPS was inhibited by DHT and NOX4 siRNA. Increased LOX-1 expression was suppressed by PDTC, a NF-κB inhibitor. Thus, NOX4 mediated the downregulation of LOX-1 expression by DHT through NF-κB.

Ox-LDL uptake and monocyte-endothelial cells adhesion play important roles in promoting atherosclerosis (Mestas and Ley, 2008; Di Pietro et al., 2016). Binding and endocytosis of ox-LDL is considered the primary function of LOX-1 in endothelial cells (Sawamura et al., 1997) and macrophages (Li et al., 2009). LOX-1 also act as an important adhesion molecule leading monocyte-endothelial cell adhesion (Honjo et al., 2003; Bai et al., 2005). LPS-induced ox-LDL uptake and THP-1 cells adhesion were inhibited by anti-LOX-1 antibody suggesting the key roles of LOX-1. The inhibitory effect of NOX4 siRNA, PDTC, and DHT provide evidence that DHT inhibited ox-LDL uptake and monocyte adhesion by downregulating LOX-1.

To further confirm the anti-atherosclerotic effect of DHT, a high fat diet induced atherosclerosis was established in ApoE^-/-^ mice. Imbalance of lipid profile, formation of atherosclerotic plaque and occurrence of necrotic cores are important characteristics of atherosclerosis (Ku et al., 1985; Shahar et al., 2003; Cai et al., 2005). Oral administration of DHT for 12 weeks dramatically decreased the serum levels of TG, TC, and LDL-C suggesting that DHT might benefit atherosclerosis by decreasing lipid levels. The decreased MDA and increased SOD and GSH by DHT also suggested that it also regulated oxidative stress *in vivo*. The reduction of atherosclerotic plaque sizes and the shrinkage of necrotic core areas in DHT treated ApoE^-/-^ mice provides the direct evidence for its anti-atherosclerotic effect. Furthermore, consistent with the *in vitro* results, expression of LOX-1, NOX4, and NF-κB in the aorta was also reduced by DHT. Thus, DHT showed anti-atherosclerotic effect in animal model.

## Conclusion

**FIGURE 9:**
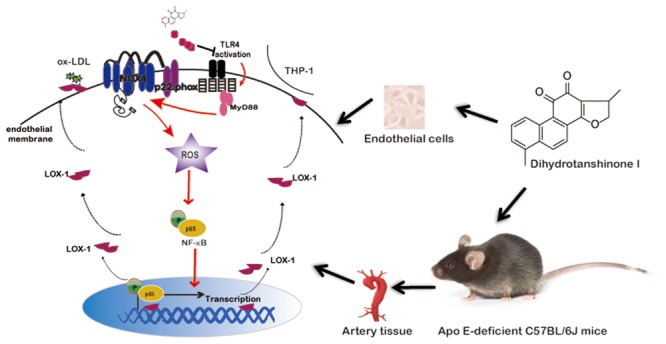
**Schematic diagram illustrating the molecular mechanisms underlying the anti-atherosclerotic effects of DHT**.

In summary, as depicted in **Figure 9**, this study showed that natural product DHT inhibited NF-κB mediated LOX-1 expression both *in vivo* and *in vitro* through NOX4-derived ROS generation. In view of the key roles of LOX-1 in the pathogenesis of atherosclerosis, inhibition of LOX-1 contributed to the anti-atherosclerotic effect of DHT.

## Author Contributions

WZ and CL are responsible for the execution of most of the experiments. HG was responsible for parts of the Western blotting. QW and JS provided critical inputs for the experiments. XC was responsible for the conceptualization, experimental design and troubleshooting, preparation of the manuscript and the financial support.

## Conflict of Interest Statement

The authors declare that the research was conducted in the absence of any commercial or financial relationships that could be construed as a potential conflict of interest.

## Footnotes

1 http://www.biovision.com/

## Abbreviations

AA: antimycin A
All: allopurinol
ApoE^-/-^ mice: apolipoprotein E-deficient mice
CMC-Na: carboxymethyl cellulose sodium
DCFH_2_-DA: 5-(6)-carboxy-2′, 7′-dichlor- odihydrofluorescein diacetate
DHE: dihydroethidium
DHT: dihydrotanshinone I
DPI: diphenyleneiodonium chloride
DPPH: 2, 2-diphenyl-1-picrylhydrazyl
GSH: glutathione
HCD: high cholesterol diet
HDL-C: HDL cholesterol
H&E: haematoxylin and eosin
HUVECs: human umbilical vein endothelial cells
ICAM-1: intercellular adhesion molecule-1
LDL-C: LDL cholesterol
LOX: lipoxygenase
LOX-1: lectin-like oxidized low-density lipoprotein receptor-1
LPS: lipopolysaccharide
MDA: malondialdehyde
MTT: 3-(4, 5-dimethyl-2-thiazolyl)-2, 5-diphenyl-2H-tetrazolium bromide
NAC: *N*-acetyl-L-cysteine
NDGA: nordihydroguaiaretic acid
NOX4: NADPH oxidase 4
N.S: no significant differences
ox-LDL: oxidized low-density lipoprotein
PDTC: pyrrolidine dithiocarbamate
ROS: reactive oxygen species
Rot: rotenone
SOD: superoxide dismutase
TC: total cholesterol
TG: triglyceride
TTFA: 2-thenoyltrifluoroacetone
XO: xanthine oxidase
