# Supplementary material for: Dihydrotanshinone I Attenuates Atherosclerosis in ApoE-Deficient Mice: Role of NOX4/NF-κB Mediated Lectin-Like Oxidized LDL Receptor-1 (LOX-1) of the Endothelium
Source: Front Pharmacol. 2016 Nov 8;7:418. doi: 10.3389/fphar.2016.00418 (PMC5105322; doi:10.3389/fphar.2016.00418)
Supplement: Supplementary file 1 [file Image_1.PDF]

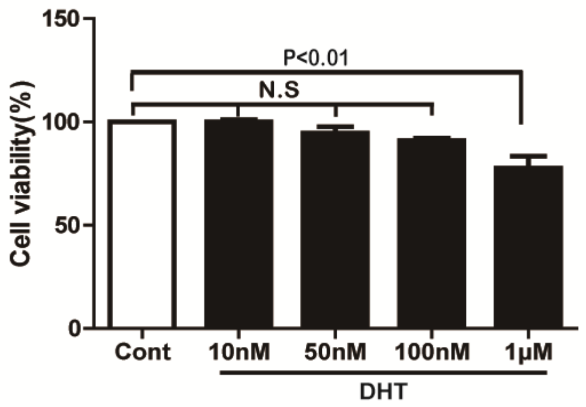

S Fig. 1 Effect of DHT on endothelial viability

Cells were treated with DHT for 24 h and the cell viability was detected by MTT assay. DHT, dihydrotanshinone I; N.S, no significant differences.

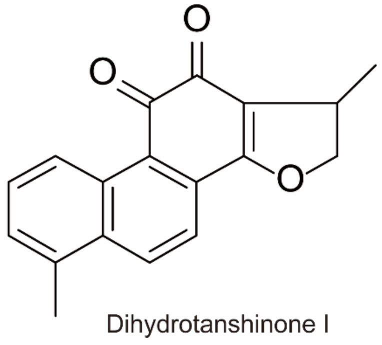

S Fig. 2 The structure of Dihydrotanshinone I

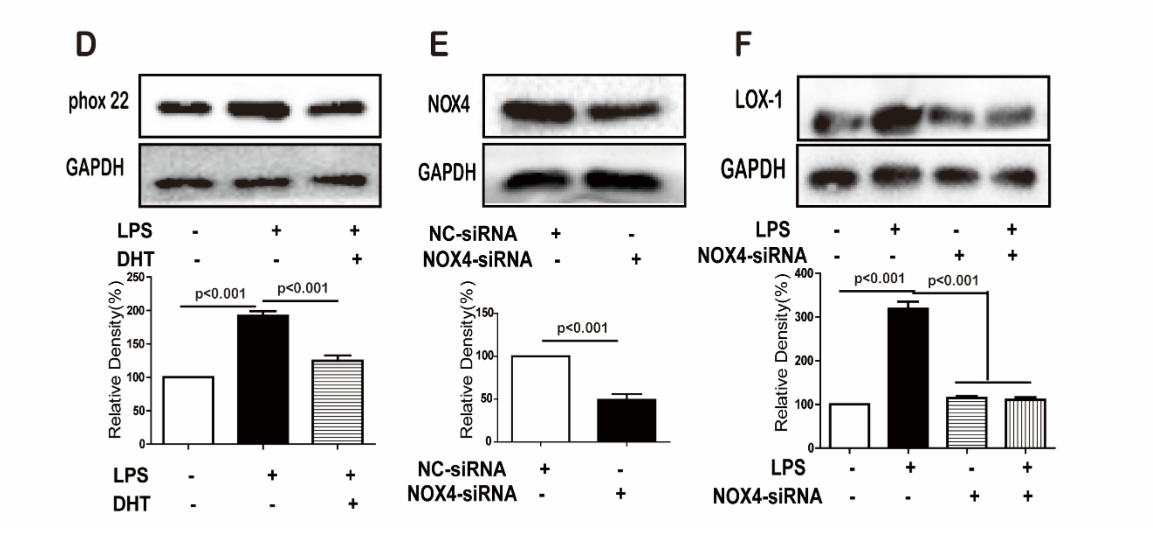

S Fig. 3 The statistical analysis of Figs. 4D, E and F

Cells were transfected with NOX4 siRNA or pretreated with DHT. Then cells were stimulated with LPS, phox 22(D), NOX4 (E) and LOX-1 expression (F) were determined by western blotting. DHT, dihydrotanshinone I; NC-siRNA, negative control siRNA;
